# Protocol for the ENCODE trial: evaluating a novel online depression intervention for persons with epilepsy

**DOI:** 10.1186/s12888-017-1229-y

**Published:** 2017-02-07

**Authors:** Björn Meyer, Mario Weiss, Martin Holtkamp, Stephan Arnold, Katja Brückner, Johanna Schröder, Franziska Scheibe, Yvonne Nestoriuc

**Affiliations:** 1 Research Department, Gaia Group, Gertigstr. 12-14, 22303 Hamburg, Germany; 2 Department of Psychology, City, University of London, London, UK; 3 Ashridge Business School, Berkhamsted, UK; 4 Department of Neurology, Epilepsy-Center Berlin-Brandenburg, Charité - Universitätsmedizin Berlin, Campus Charité Mitte, Charitéplatz 1, 10117 Berlin, Germany; 5 Schön Clinic Vogtareuth, Clinic for Eplilepsy, Krankenhausstraße 20, 83569 Vogtareuth, Germany; 6 Department of Neurology and Epileptology, Epilepsy-Center Hamburg, Evangelical Hospital Alsterdorf, Elisabeth-Flügge-Straße 1, 22337 Hamburg, Germany; 7 Department of Psychosomatic Medicine and Psychotherapy, University Medical Center Hamburg-Eppendorf and Schön Klinik Hamburg Eilbek, Martinistraße 52, 20246 Hamburg, Germany; 8 Department of Psychiatry and Psychotherapy, University Medical Center Hamburg-Eppendorf, Hamburg, Germany

**Keywords:** Epilepsy, Depression, Anxiety, Internet interventions

## Abstract

**Background:**

Depression is common among persons with epilepsy (PwE), affecting roughly one in three individuals, and its presence is associated with personal suffering, impaired quality of life, and worse prognosis. Despite the availability of effective treatments, depression is often overlooked and treated inadequately in PwE, in part because of assumed concerns over drug interactions or proconvulsant effects of antidepressants. Internet-administered psychological interventions might complement antidepressant medication or psychotherapy, and preliminary evidence suggests that they can be effective. However, no trial has yet examined whether an Internet intervention designed to meet the needs of PwE can achieve sustained reductions in depression and related symptoms, such as anxiety, when offered as adjunct to treatment as usual.

**Methods/Design:**

This randomized controlled trial will include 200 participants with epilepsy and a current depressive disorder, along with currently at least moderately elevated depression (Patient Health Questionnaire (PHQ-9) sum score of at least 10). Patients will be recruited via epilepsy treatment centers and other sources, including Internet forums, newspaper articles, flyers, posters, and media articles or advertisements, in German-speaking countries. Main inclusion criteria are: self-reported diagnosis of epilepsy and a depressive disorder, as assessed with a phone-administered structured diagnostic interview, none or stable antidepressant medication, no current psychotherapy, no other major psychiatric disorder, no acute suicidality. Participants will be randomly assigned to either (1) a care-as-usual/waitlist (CAU/WL) control group, in which they receive CAU and are given access to the Internet intervention after 3 months (that is, a CAU/WL control group), or (2) a treatment group that may also use CAU and in addition immediately receives six-month access to the novel, Internet-administered intervention. The primary outcome measure is the PHQ-9, collected at three months post-baseline; secondary measures include self-reported anxiety, work and social adjustment, epilepsy symptoms (including seizure frequency and severity), medication adherence, potential negative treatment effects and health-related quality of life. Measurements are collected online at pre-treatment (T0), three months (T1), six months (T2), and nine months (T3).

**Discussion:**

Results of this trial are expected to extend the body of knowledge with regard to effective and efficient treatment options for PwE who experience elevated depression and anxiety.

**Trial registration:**

ClinicalTrials.gov: NCT02791724. Registered 01 June 2016.

## Background

Among persons with epilepsy (PwE), depression is exceedingly common and is associated with intense personal suffering and dramatic reductions in quality of life [1]. According to a recent systematic review, 23.1% of PwE are affected by depression within the past year [2], compared to 6.6% in the general population [3]. Lifetime depression prevalence is estimated at 30 to 35 percent among PwE [4], compared to 16.2% in the general population [3].

Even though their frequent co-occurrence has been observed repeatedly, questions remain regarding the causal mechanisms underlying the relationship between epilepsy and depression. The conventional view has long held that depression arises as a consequence of having epilepsy, but evidence is not consistent with such unidirectional causation [4–7]. Indeed, experiencing depression before seizure onset may increase risk for the subsequent development of epilepsy and, vice versa, having epilepsy may increase subsequent depression risk [5]. Research suggests that multiple pathogenic mechanisms might explain the common co-occurrence of epilepsy with depression and related syndromes, such as anxiety [4]. These include common neurobiological pathways, including neurotransmitter disturbances, brain-structural or neuropathological abnormalities, and psychosocial pathways, such as inadequate social support, stigma or maladaptive coping [1, 4, 6, 8]. Additionally, iatrogenic pathways must also be considered, as some antiepileptic drugs facilitate depression and anxiety symptoms [6].

Research has attempted to identify sociodemographic and disease-related risk factors for depression and anxiety among PwE, albeit with limited success [9]. According to a recent systematic review, age and gender are not associated consistently with depression, whereas seizure frequency and recency appear to increase depression risk [9]. Different seizure types, however, are not linked differentially with depression or anxiety. Overall, it remains difficult to predict the course of psychiatric symptoms among PwE from known patient characteristics, even though an increased general risk has been established, compared to healthy comparison populations.

Several pharmacological and psychosocial depression and anxiety treatments have been shown to be effective among PwE. A recent systematic review reported that cognitive behavioral therapy (CBT) might help alleviate depression among PwE, although it appears to be less effective for seizure control, and more high-quality studies are needed before definitive conclusions can be drawn [10]. Even though CBT and perhaps other forms of psychotherapy are promising treatments for anxiety or depression among PwE, they require the availability of trained therapists, ideally with experience in treating PwE, which is often unrealistic in many treatment settings. According to an international expert consensus statement, CBT as well as psychotropic medication (particularly selective serotonin reuptake inhibitors) are recommended for the treatment of depression and anxiety in PwE [11]. Unfortunately, though, the vast majority of depressed PwE typically do not receive any depression-related treatment; in a recent study, depression remained untreated in 70% of depressed PwE [12].

Even though treatments with some degree of effectiveness exist, then, depression and anxiety often remain undetected and undertreated among PwE, for at least two reasons: (1) Epilepsy symptoms can overlap with or mask psychiatric symptomatology, making detection difficult and ambiguous (e.g., fatigue can be a symptom of epilepsy, depression, or both), and (2) physicians are often reluctant to prescribe antidepressants because of concerns over side effects, drug interactions, or lowered seizure thresholds [13]. Additionally, other barriers may prevent depressed individuals from seeking or accessing depression treatment, including stigma concerns, time constraints, lack of motivation, skepticism regarding psychotherapy, disease-related restrictions (e.g., inability to drive), and perceived lack of necessity [14]. It is important, though, to identify depression, overcome potential barriers, and initiate treatment, as untreated depression in PwE may increase risk for work absenteeism, increased health care system utilization, and direct medical costs [15].

To improve access and extend the range of available depression treatment options, Internet-based treatments could play a key role [16, 17]. Indeed, several Internet-based interventions have been developed and tested over the past decade and are making a considerable impact upon the field of mental health care delivery [18, 19]. As early as 2002, expert panels have recommended exploiting the Internet to deliver evidence-based psychological treatments to underserved populations [20], and recent systematic reviews and meta-analyses have confirmed that some such interventions, most of which are based on CBT-principles, are effective for a range of psychiatric symptoms and conditions, including depression and anxiety disorders [21–23]. However, this body of research has also been criticized: many trials are conducted solely online, without establishing contact with participants to verify identity and diagnoses, and they suffer from various methodological problems such as excessive attrition and lack of follow-up data [24]. Nevertheless, at least in some countries, including Sweden, the Netherlands, Australia, and the United Kingdom, Internet-based psychological interventions with robust evidence are finding their ways into national treatment guidelines and are increasingly integrated in routine care services for patients suffering from depression, anxiety, and other psychiatric conditions [18, 25, 26]. Despite the considerable promise and success of some Internet-based psychological interventions, though, they have rarely been applied to the treatment of depression and anxiety among PwE.

To our knowledge, only one randomized controlled trial to date has examined whether an Internet-administered intervention can reduce depression among PwE [27]. In this study, Schröder et al. randomized 78 PwE either to a care-as-usual (CAU) control or to an intervention condition, in which participants received CAU plus access to a depression-focused Internet intervention termed Deprexis, which has been tested in non-epilepsy samples in seven additional studies [28–34]. In the Schröder et al. trial, participants in the intervention group experienced significantly greater depression reduction than those in the control group over the course of nine weeks, with a small to moderate post-treatment between-groups effect size (Cohen’s *d* = .43). The study suffered from some methodological limitations, though, including lack of long-term follow-up data and structured diagnostic interviews. Perhaps most importantly, the intervention was not tailored to address the unique needs of PwE, as noted by the authors: “In their subjective appraisal … most participants found that the program should be adapted to the special needs of PwEs with respect to involving more epilepsy-related topics, [which] … could increase the acceptability as well as the effectiveness of the intervention in this particular patient group” [27]. The Deprexis program also does not target anxiety, which is a common comorbid condition among PwE and correlates as highly as *r* = .75 with depression [1, 6, 35], justifying the development of interventions for both syndromes rather than just one.

The goal of the present randomized controlled trial (RCT) is to test the efficacy of a novel CBT-based, depression-focused Internet intervention that specifically addresses the needs of PwE. This new program was developed by the same group of therapists and researchers that developed Deprexis, the above-mentioned intervention that was shown to be effective in the treatment of depression among PwE. The novel intervention has been developed and is operated by *Gaia*, an e-Health company with more than ten years of experience in the development of e-Health interventions, located in Hamburg, Germany. The content and technical design of this intervention are described in the methods section below.

The goal of this parallel-groups, pragmatic RCT is to evaluate the extent to which a novel Internet intervention, used adjunctively to CAU, can contribute to improving symptoms of depression and anxiety, social-occupational functioning, physical health (epilepsy symptoms), medication adherence, and health-related quality of life, among PwE. Furthermore, the trial aims to evaluate the extent to which patients with epilepsy regard this intervention as a helpful and valuable tool. The primary focus of the software-based intervention is on facilitating depression reduction; hence, this is the target identified in the primary hypothesis.

It is hypothesized that, between baseline and three months, patients randomized to the intervention group will report greater reductions in depression, as measured by the Patient Health Questionnaire (PHQ-9), a well validated depression measure [36, 37], compared to patients randomized to the control condition. The goal of this pragmatic RCT [38] is to test the effectiveness of a novel Internet intervention on depression reduction among PwE over a period of three months, in comparison to a CAU/waitlist (CAU/WL) control group, which only receives access to the intervention after three months. Data will also be collected at six and nine months, which will allow us to examine the stability of intervention effects over time.

Secondary measures are administered to examine effects of the intervention on anxiety symptoms, stress symptoms, and depression-related psychosocial impairment, and it is hypothesized that participants assigned to the intervention group will show greater improvements in these respective measures over three months, compared to control group participants. Exploratory analyses will be conducted to examine potential negative effects of the intervention, effects on epilepsy symptom severity and seizure frequency, health-related quality of life, epilepsy self-management, and medication adherence. The subjective usefulness of the intervention will also be examined, and we expect that users will rate the intervention favorably, given that it was developed to address several of their epilepsy-specific needs and concerns.

## Methods/Design

### Study design

**Fig. 1:**
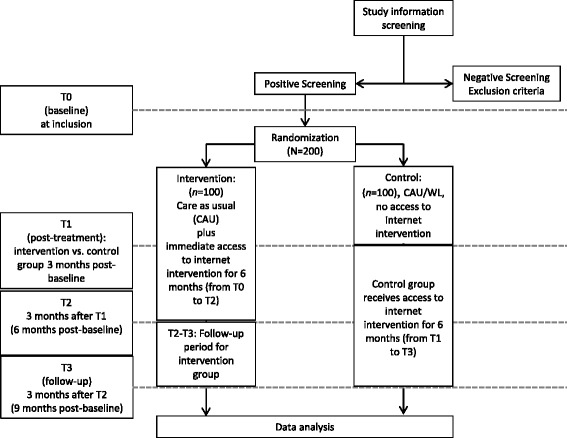
Flow diagram of the randomized controlled trial

The content of this RCT as well as the design is in accordance to the guidelines for clinical trial protocols as specified the by the SPIRIT 2013 statement [39]. Moreover, this RCT is registered in ClinicalTrials.gov (NCT02791724). Any changes to this trial protocol will be described in this trial registry. Patients will be randomized to two groups: (1) a control group, in which they may engage with any epilepsy treatment and receive access to the Internet intervention after a delay of three months (CAU/WL), or (2) to a treatment group that immediately receives six-month access to the Internet intervention and may also use CAU (see Fig. 1 for flow chart). Patients will be recruited consecutively from various sources, including epilepsy treatment centers and outpatient clinics, with every patient having an equal chance of being assigned to the intervention or control group (no block randomization). Randomization will be performed by the Principal Investigator (PI), using a computer-generated sequence to generate the allocation sequence. Participants will be enrolled by trained research associates; the allocation sequence will be concealed from them. Given the pragmatic design, participants are not blinded as to group assignment.

Consistent with the logic governing pragmatic RCTs, the trial will examine whether utilizing this program will improve depression outcome above and beyond the routine care patients are actually receiving [38, 40, 41]. Furthermore, particular attention will be paid to potential negative effects of the program, as assessed by a questionnaire. The trial thus aims to maximize external validity by contrasting the realities of current clinical care with the addition of an Internet intervention; it does not aim to maximize internal validity by selecting a maximally homogeneous patient group or introducing many other artificial control measures, such as narrow inclusion and exclusion criteria that do not reflect the clinical reality outside of trials [40].

### Recruitment and assessment

Participants will be recruited from various settings, including epilepsy treatment clinics, outpatient treatment centers, epilepsy support groups, Internet forums and groups, or Facebook pages. Methods such as newspaper articles, flyers, posters, and media articles or web-advertisements will be used to inform potential participants about the study (all material will be in German). Treatment centers are informed about the goals and procedures of the study; they were provided with information material (study flyers, posters or leaflets) that can be distributed to patients. This information material was scrutinized by the locally responsible ethics board prior to study commencement. Patients who are interested in participating are invited (in the printed study material) to sign up with their name and e-mail address at an online study website (https://de.research.net/r/epilepsie-studie). A member of the research team will then contact the potential participant by e-mail within one week in order to invite the person to the online baseline assessment (T0). Part of this online baseline assessment is to provide further information about the study as well as to provide informed consent to participate (the informed consent form in German is available on request from the first author). After completing the T0 online assessment and provided that informed consent is given, the research team associate will then conduct a telephone interview in order to check inclusion/exclusion criteria and confirm the diagnosis of a depressive disorder with a structured diagnostic interview (the MINI, as used in previous studies by this group [32, 42, 43]). After the telephone interview, patients will be randomized and will be asked to complete 3-months (T1, post-treatment), 6-months (T2), and 9-months (T3) online assessments (see Table 1). The T1 (post-treatment) assessment will permit us to examine whether treatment response can be documented after this relatively brief period, as has been shown in previous trials with a similar intervention [27–32]. The T1 assessment will be the time-point at which the intervention group but not the control group has used the intervention, thus permitting us to test the primary hypothesis that depression reduction will differ over three months between the intervention versus control groups.

**Table 1:** Study measures and measurement points

	MINI^a^	PHQ-9^b^	NDDIE^c^	GAD-7^d^	DASS-21^e^	WSAS^f^	Subjective usefulness of the program	INEP^g^	Epilepsy symptom severity^h^	Medication adherence^i^	Health-related quality of life^j^	ESMS^k^
T0 baseline	X	X	X	X	X	X			X	X	X	X
T1 (3 months post-baseline)		X	X	X	X	X	X^l^	X^l^	X	X	X	X
T2 (6 months post-baseline)		X	X	X	X	X	X	X^m^	X	X	X	
T3 (9 months post-baseline)		X	X	X	X	X	X^m^		X	X	X	

^a^MINI Neuropsychiatric Interview

^b^Patient Health Questionnaire

^c^Neurological Disorders Depression Inventory for Epilepsy

^d^Generalized Anxiety Disorder questionnaire, 7 items

^e^Depression and Anxiety Scales

^f^Work and Social Adjustment Scale

^g^Inventory for the Assessment of Negative Effects of Psychotherapy

^h^PESOS and Liverpool Seizure Severity Scale (LSSS)

^i^Rief Adherence Index (RAI)

^j^QOLIE-10-P (Quality of Life Inventory in Epilepsy)

^k^Epilepsy Self-Management Scale

^l^Only patients in the intervention group

^m^Only patients in the control group

### Inclusion criteria

The inclusion criteria for this trial are as follows:

1. Age at least 18 years,
2. diagnosis of active epilepsy (that is, at least one unprovoked epileptic seizure within the past 10 years or taking antiseizure medication within the past five years in the context of epilepsy [44]),
3. current depressive disorder (either major depressive disorder or dysthymic disorder, as determined by telephone interview with the MINI [43]),
4. currently at least moderate self-reported depressive symptom severity, as operationalized by a score of at least 10 on the PHQ-9, a cut-off score that has been well validated [45, 46],
5. ability to speak and read German,
6. access to the Internet and personal possession of an appropriate device on which the Internet-based intervention can be used regularly (e.g., modern smartphone, computer, laptop),
7. motivation to participate in the trial and use an Internet-based intervention to acquire skills and knowledge that may aid in the amelioration of depression symptoms.

### Exclusion criteria

Exclusion criteria are as follows:

1. Newly prescribed antidepressant medication or changes in antidepressant dosage during the one month prior to study inclusion (medication changes after study inclusion are permitted, given the pragmatic design of the trial),
2. currently in psychotherapy,
3. presence of bipolar disorder, schizophrenia or another psychotic disorder, or borderline personality disorder (based on the MINI interview),
4. presence of acute suicidality (that is, no intention or plan to commit suicide, as assessed with the respective module of the MINI),

### Intervention

The Internet-based intervention evaluated in this trial was designed with the aim of conveying evidence-based psychotherapeutic techniques, based on CBT, to PwE over a period of 180 days. The intervention is fully self-guided (i.e., no guidance or support from a clinician is provided) and focuses on identifying and challenging cognitions that trigger or sustain depression and anxiety, increasing activities that are likely to reduce depression and anxiety, practicing relaxation and stress management exercises and increasing mindfulness skills (see below for a more detailed content description). The program is based on general CBT, evidence-based psychotherapeutic techniques that have been shown to be helpful for patients suffering from depression (and ideally, that have been shown to be helpful among PwE). Furthermore, program content addresses other important issues in disease self-management, including lifestyle habits (e.g., nutrition, exercise) and medication adherence. The delivery and training of content is continuously individualized to match users’ preferences and needs, based on responses within the program. Epilepsy patients are guided through the program by rule-based artificial intelligence algorithms that use patient responses as input. The intervention is delivered via the Internet and protected by individually assigned passwords.

In the development process, several steps were taken to ensure the quality and safety of the software: (1) The program was developed by an experienced team consisting of clinical psychologists, CBT therapists, physicians, software engineers, graphic artists, speakers, and sound engineers, among others. This team has already developed several other programs with demonstrated safety and efficacy in clinical trials [28, 32, 47–51], (2) volunteers (PwE) were involved continuously throughout the development process to test preliminary versions and provide feedback, consistent with development guidelines [52, 53], (3) expert feedback was sought from several physicians specializing in the treatment of epilepsy, who reviewed the program, participated in informal discussion rounds with the developers, and provided written feedback. Additionally, literature on previous CBT developments among PwE was reviewed [10, 54–56]. The development of the program took place over a period of approximately one year in 2015.

The intervention was designed to be fully functional on current generation smartphones (e.g., iPhone 6 and 7) as well as on technologically simpler, older, low-cost smartphones that have Internet connectivity. Moreover, the intervention can be used on smartphones as well as larger-screen devices (tablet-PCs, laptops, desktop PCs), with seamless integration and continuity for individual users (e.g., users can freely switch back and forth among different devices, always continuing where they left off, or find features that accumulate over time on any given device, provided that they log on with their unique user-ID). Like other Internet interventions developed by this group, the software-based intervention uses cloud computing with fast global access and is securely hosted in an ISO-27001-certified data center located in Germany. The systems use a CE-certified platform technology, and other programs developed by this group, such as Deprexis [28, 32, 49], are certified as medical devices across the European Union.

Like other programs developed by this group, the intervention described herein is produced on a proprietary software (broca®) that was developed by *Gaia* in the early 2000s and is currently in its fourth iteration. This software is designed to allow for extensive tailoring of content to match both stable and dynamically changing user requirements, consistent with evidence showing the superiority of tailored over generic health behavior interventions [57]. The programs engage users in simulated “dialogues” of varying length in which brief content chunks are continuously followed by response options. Depending on which responses are selected, subsequent content is altered to match emerging preferences or requirements - analogous to the type of “responsiveness” that effective psychotherapists use to change micro-interventions throughout the course of treatment [58]. Tailoring may enhance the personal relevance of information and lead to enhanced cognitive activity and information retrieval, which may explain, to an extent, the superior effects of tailored interventions [59].

An outline of main content and program features is provided below. Each of five modules contains content that can be explored in briefer or longer individual sessions, depending on personal preferences. Depending on factors such as reading speed, interest to explore content in greater or lesser depth, and desire to listen to a greater or smaller number of audio recordings, each module can be completed in approximately 60 to 180 min. Thus, it would be possible to work through the entire program in as little as approximately five hours, or one could choose to spend much more time with it. As in other programs developed by our group [27–34, 48], there is no fixed or generic sequence in which modules must be completed, nor a minimum number of sessions that must be finished. Users are invited to freely explore the program and let themselves be guided within each section by the algorithm-driven sequences generated by the program. They are also informed that they can discontinue the program at any time if they feel that the program is not helpful or even harmful, and they are invited to contact the Principal Investigator, a licensed clinical psychologist, if they seek further help or advice. The purpose of such contacts would be only to provide information regarding treatment options, if clinically required, but not to provide therapeutic support. To define minimally sufficient usage, we are using the same algorithm we have used previously [32]: Having started at least four sessions and spent a total of least 60 min actively engaged with the program.

Program elements:

1. *Introductory module:* The introductory module provides an overview of the purpose, functions, and time-frame of the program. Patients are engaged in interactive sequences in which the CBT approach is briefly explained and in which their current and past medical and psychiatric history are explored. The introductory module addresses topics such as epilepsy onset and severity, types and intensity of current depression and anxiety symptoms, as well as exercise and nutrition habits. Patients are provided with a summary (“personal profile”) of their responses, and recommendations are made for further topics that patients may wish to engage with in subsequent sessions. The recommendation is made that patients use the program several times per week, as they see fit, for at least three months. They are informed that program access is provided for a six-month period.
2. *Depression module:* The depression module is individualized in such a manner that patients can continuously explore topics in greater or lesser depth and are presented with content that is adapted to their previously expressed concerns and needs. The module is structured, on a broad level, in two separate sections: (a) a psychoeducational part in which topics such as depression symptoms and treatment options can be explored interactively, and (b) an intervention part in which CBT-based techniques, such as identifying negative automatic thoughts and cognitive distortions as well as challenging or refuting unhelpful thoughts (cognitive modification), can be learned and practiced. In the latter part, patients learn about the nature and functions of negative automatic thoughts and cognitive distortions, they are presented with a rationale for activity scheduling and the principles of behavioral activation, they are offered several mindfulness and acceptance exercises, and they have the option of engaging with *cognitive bias modification for interpretation* techniques [60–62]. As in all other parts of the program, references and suggestions for further information are provided.
3. *Anxiety module:* The anxiety module is broadly consistent with contemporary CBT and transdiagnostic approaches to anxiety [63–65], which have already been shown by our group to be effective when delivered via an Internet intervention [48]. Interactive sequences are used to explain the nature and function of anxiety, emphasizing the idea that anxiety can serve a useful function (e.g., signaling potential danger) but can be unhelpful when it is automatically triggered easily in the absence of actual danger (i.e., “false alarm” model). Core CBT principles are explained, such as exposure, avoidance behavior, cognitive restructuring, acceptance of aversive emotions, willingness to pursue valued goals even in the presence of anxiety, and mindfulness and relaxation exercises. Content is focused on concerns relevant for PwE, such as experiencing future seizures or worsening symptoms, not being able to drive, not functioning on the job, experiencing side effects, social embarrassment, or becoming a burden for others [66, 67]).
4. *Coping with epilepsy symptoms module*: This module focuses on several epilepsy-specific topics, including (a) medication adherence (e.g., exploring motivation with regard to medication-taking, using decisional balance exercises in which perceived advantages and disadvantages of taking medications are weighed, fostering self-efficacy [68]), (b) identifying and coping with seizure auras and triggers [55, 69, 70], (c) dealing with stigma and discrimination [71], (d) reflecting on values and life goals that are realistic and attainable even with a condition such as epilepsy [72, 73].
5. *Lifestyle modification module:* This module focuses on topics such as the role of exercise and dieting/nutrition in the management of epilepsy. There is persuasive evidence that both exercise and healthy nutrition affect depression and anxiety, both in healthy populations and among those with chronic illnesses [74–78]. Epilepsy-specific topics are explored interactively in some depth, such as the potential utility of adopting a ketogenic diet [79] and the advantages and risks of different types of exercise [80].
6. *Symptom tracking:* Self-monitoring of symptoms is an essential aspect of CBT that is particularly suitable for mobile interventions [81] and has been integrated successfully in previous Internet interventions developed by our group [48, 49]. In this intervention, items from a validated questionnaire [82] that assesses the severity of depression and anxiety symptoms are integrated. Patients are invited to complete the items at regular intervals and track their symptom severity via visual and text-feedback that is provided in the program.

Similar to other Internet interventions developed by our group, this intervention also includes the following elements: (a) optional daily text messages (either via SMS or e-mail) for 90 days, in which brief motivational content is conveyed, (b) worksheets and brief summaries of module content, (c) audio recordings within each module. Program usage is tracked automatically by the software.

### Outcome measurements

All outcome measurements will be collected via a secure, encrypted online survey service. Participants will be invited via e-mail to complete the online self-report measures, and up to two reminder e-mails will be sent if they do not respond to the initial invitation.

#### Primary outcome

The primary outcome, symptoms of depression, will be measured at T0, T1, T2, and T3 by the PHQ-9, a well-validated measure of depressive symptom severity [36, 37]).

#### Secondary outcomes

The following instruments will be used to assess secondary outcomes. Each instrument will be administered at T0, T1, T2, and T3, except of the Inventory for the Assessment of Negative Effects of Psychotherapy (INEP), the items regarding the subjective usefulness of the program and the Epilepsy Self-Management Scale (ESMS).

1. The *NDDIE (Neurological Disorders Depression Inventory for Epilepsy* [83]*)* will be used as a secondary measure of depression symptom severity.
2. *GAD-7 (Generalized Anxiety Disorder questionnaire, 7 items)* [84, 85]: The GAD-7 is a well-validated measure of anxiety symptom severity. Originally developed to assess symptoms of generalized anxiety disorder, it has been shown to be a valid and reliable measure of anxiety severity more broadly conceptualized.
3. *DASS-21 (Depression and Anxiety Scales)* [82]: The DASS-21 is a valid and reliable brief questionnaire of depression, anxiety, and stress symptoms.
4. *WSAS (Work and Social Adjustment Scale)* [86]: The WSAS is a validated brief index of psychosocial impairment caused by depression (e.g., inability to work or pursue hobbies due to depression). The WSAS has been used in previous research by this group; psychometric properties have been found to be adequate [28].
5. *Subjective usefulness of the program:* measured by individually designed items, as in previous studies by this research group [28, 29, 32]. The items will be administered after using the intervention (intervention group: T1 and T2, control group: T2 and T3).
6. *INEP*: INEP is a reliable self-report instrument for assessing potential negative effects of psychotherapeutic treatment. A version that has been adapted to inquire about potential negative effects of Internet interventions is used here [87]. The INEP will be administered after 3-month using the intervention (intervention group: T1, control group: T2).
7. *Epilepsy symptom severity:* measured by validated measures (e.g., seizure frequency and severity; PESOS (Performance, Sociodemographic aspects, Subjective evaluation) [88] and LSSS (Liverpool Seizure Severity Scale) [89]).
8. *Medication adherence:* Measured by a brief, validated questionnaire, such as the 4-item Rief Adherence Index (RAI) [90].
9. *Health-related quality of life:* Measured with a validated questionnaire that assesses quality of life among PwE, the QOLIE-10 (Quality of Life Inventory in Epilepsy) [91].
10. *ESMS*: The ESMS is a reliable questionnaire that assesses frequency of use of epilepsy self-management practices [92]. The ESMS will be administered at T0 and T1.

Additionally, several items will be administered at the relevant time-points to assess specific demographic characteristics (e.g., age, gender) and illness-related parameters including:

- Frequency of interfering seizures (at T0):
  - “Did you experience at least one seizure within the past year?” (yes, no)
  - If no: “When did you last experience a seizure that interfered with your ability to pursue your normal activities?”
  - “How often are you currently typically experiencing seizures that interfere with your ability to pursue your normal activities?” (no seizures, 1–5 seizures per month, 6–10 seizures per month, > 10 seizures per month)
- Seizure frequency at subsequent time-points (at T1, T2, T3):
  - “Did you experience at least one seizure within the past three months?” (yes, no)
  - If yes: “Over the past three months, how many seizures have you experienced that interfered with your ability to pursue your normal activities?”
- Current medication (at all time-points):
  - “Which seizure medicine(s) are you currently taking? (please list)”

### Sample size calculation and analysis

The sample size of this study is based on the expected difference on the primary outcome variable (depressive symptom severity), between the intervention group and the control group at T1 (three months). Based on a power of at least 0.80 in a two-tailed test and an alpha of 0.05, randomization of 200 subjects (100 per group) will be sufficient to show an effect-size of *d* = 0.50 (moderate effect), anticipating an attrition rate of maximally 20% at T1, a rate that has been achieved in several previous trials [29–32]. With an anticipated sample size of 160 (2 × 80) completers at T1, power of .88 would be achieved for a moderate effect size of *d* = 0.50. Based on previous research by this group [27–29, 32], the assumption of achieving a moderate effect appears realistic. This power calculation is based on the assumption that an even randomization procedure (50:50) will be used.

Analyses will conform to recommended methodological standards, as specified by the CONSORT statement [93]. Specifically, both intention-to-treat and per-protocol analyses will be conducted, using appropriate methods such as linear mixed-models, which are widely used in this field and have been recommended because of their capacity to handle missing data appropriately [94, 95]. It is anticipated that attrition rate at post-treatment will be below 20%, given previous trial experiences [29, 31, 32]. Additional analyses will be performed to examine the potential influence of confounding variables on changes in the primary outcome (e.g., initial differences in symptom severity or treatment utilization).

### Conflict of interest, scientific integrity and independence

It is not unusual for intervention developers to be involved in trials that examine their efficacy; indeed, this is the rule rather than the exception and will also be the case in this study. However, measures will be taken to ensure that potential conflicts of interests do not jeopardize the scientific integrity of data collection, analysis, or any other aspect of the study. Firstly, the Principal Investigator (YN) is an independent established researcher in the field of clinical psychology and psychotherapy without financial or other ties to the developers, operators, and sponsors of this project (i.e., YN does not receive remuneration or other compensation from Gaia, the developers). Secondly, the Principal Investigator and members of her research team will have full and continuous access to all data being collected in this study (provided, of course, that informed consent to this is granted by participants at study commencement). Data monitoring will be performed by the Principal Investigator, who is independent from the sponsor and has no competing interests. All adverse events will be reported; interim analyses are not planned. The day-to-day management of the study (e.g., contacting participants and inviting them to complete online surveys) will be performed by research associates working at Gaia, the intervention developer, which is an e-Health enterprise with an established track record of multicenter research [28, 32, 42, 96]. Publication of results will be sought regardless of study outcome, i.e., even if the intervention should prove to be ineffective. A summary of the results will also be made available for trial participants after completion of the trial.

## Discussion

This protocol describes a methodological rigorous, statistically adequately powered trial of a novel Internet-based psychological treatment for depression and anxiety among PwE. There is an urgent need for innovate and low-threshold interventions in this area, given that depression and anxiety are exceedingly common in epilepsy, complicate treatment response and prognosis, dramatically reduce quality of life, and yet often remain undetected and are treated inadequately. The personal suffering and societal burden associated with depression among PwE could potentially be reduced by Internet-based interventions such as the one evaluated in this trial.

Although the allegedly low costs of Internet-based interventions are often emphasized, developing and maintaining effective, technologically adequate and secure Internet interventions incurs considerable expenses. In this project, a research-focused e-Health company is driving the development of a novel Internet intervention and investing considerable resources to enable the conduct of a methodologically rigorous, independent scientific investigation, whose results will be published regardless of whether they are consistent with the research hypothesis, and the hopes or expectations of the program developers. Several collaborating investigators with no conflicts of interest will ensure the scientific integrity and independence of this project. In our view, this type of collaborative arrangement seems ethically responsible and scientifically viable. Along with others [19, 97, 98], we feel confident that evidence-based Internet interventions will increasingly be regarded as legitimate, valuable additions to the armamentarium of professional medical care, particularly if their value can be demonstrated repeatedly in rigorously designed trials.

First and foremost, this trial seeks to make a scientific contribution to the field of depression and anxiety treatment among PwE. This will be the first trial in which an Internet-based intervention is evaluated that has been designed specifically to address the unique needs of PwE. It is also one of the few such trials in which diagnoses are established by a structured, validated interview. Several additional methodological strengths are noteworthy, including follow-up assessments and the inclusion of an array of validated measures for the primary outcome of depression and secondary outcomes such as anxiety, quality of life, epilepsy disease status, and potential negative effects of treatment, which are highly relevant for the quality and safety management of novel Internet interventions. As in most studies, these strengths are balanced by some methodological limitations, including the lack of clinician outcome-ratings, laboratory tests, or brain imaging measures. Studies utilizing such methods are desirable but costly, and it is hoped that future investigations, including health-economic studies, will further explore the effects and utility of the novel, Internet-based intervention described and tested in this trial.

### Trial status

Recruitment is ongoing. It is anticipated that the trial will be completed (T3) by October 2017.

## Abbreviations

CAU: Care-as-usual
CBT: Cognitive behavioral therapy
DASS-21: Depression and Anxiety Scales
ESMS: Epilepsy Self-Management Scale
GAD-7: Generalized Anxiety Disorder questionnaire, 7 items
INEP: Inventory for the Assessment of Negative Effects of Psychotherapy
PHQ-9: Patient health questionnaire, 9 items
PwE: Persons with epilepsy
RCT: Randomized controlled trial
WSAS: Work and social adjustment scale
